# Effects of elastic band exercise on lean mass and physical capacity in older women with sarcopenic obesity: A randomized controlled trial

**DOI:** 10.1038/s41598-018-20677-7

**Published:** 2018-02-02

**Authors:** Chun-De Liao, Jau-Yih Tsauo, Shih-Wei Huang, Jan-Wen Ku, Dun-Jen Hsiao, Tsan-Hon Liou

**Affiliations:** 1 School and Graduate Institute of Physical Therapy, College of Medicine, National Taiwan University, Taipei, Taiwan; 2 Department of Physical Medicine and Rehabilitation, Shuang Ho Hospital, Taipei Medical University, Taipei, Taiwan; 3 Department of Physical Medicine and Rehabilitation, School of Medicine, College of Medicine, Taipei Medical University, Taipei, Taiwan; 4 Department of Radiology, Shuang Ho Hospital, Taipei Medical University, Taipei, Taiwan; 5 College of Public Health and Nutrition, Taipei Medical University, Taipei, Taiwan; 6 Graduate Institute of Injury Prevention and Control, Taipei Medical University, Taipei, Taiwan; 7 Graduate Institute of Sports Science, National Taiwan Sport University, Taoyuan, Taiwan

## Abstract

Sarcopenia is associated with loss of muscle mass as well as an increased risk of physical disability in elderly people. This study was aimed to investigate the effect of elastic band resistance training (ERT) on muscle mass and physical function in older women with sarcopenic obesity. A randomized controlled trial with an intention-to-treat analysis was conducted. A total of 56 women (mean ± SD age 67.3 ± 5.1 years) were randomly assigned to the experimental group receiving 12 weeks of ERT and to the control group receiving no exercise intervention. Lean mass (measured using a dual-energy X-ray absorptiometer), physical capacity (assessed using the global physical capacity score), and a 36-item short form questionnaire were conducted at the baseline examination (T_0_), as well as the 3-month (T_1_) and 9-month followups (T_2_). At T_1_ and T_2_, the between-group difference was measured in total skeletal mass relative to T_0_, with mean differences of 0.70 kg (95% CI 0.12–1.28; *P* < 0.05) and 0.72 kg (95% CI 0.21–1.23; *P* < 0.01), respectively. Similar results were found in muscle quality, physical capacity, and physical function outcomes. The ERT exerted a significant beneficial effect on muscle mass, muscle quality, and physical function in older women with sarcopenic obesity.

## Introduction

Sarcopenic obesity, a recently identified phenotype of obese elderly people, is engendered by the additive effect of sarcopenia and obesity^1^. Although sarcopenia has been termed as—and is characterized by—age-related muscle wasting^2^, obesity involving an increase in adipose tissue is also considered a major cause of skeletal muscle loss^3^. Such a decline in muscle mass followed by insufficient muscle power contributes to low physical performance in frail elderly people^4,5^. Furthermore, reports have associated obesity with physical disability^6,7^. Under these circumstances, studies have identified that sarcopenic obesity with its underlying additive effect of muscle loss is associated with a higher level of physical limitations than sarcopenia or obesity alone; moreover, sarcopenic obesity is considered a risk factor for disability and frail conditions^5,8,9^.

Muscle mass is maintained through regulation of the balance of skeletal muscle turnover between muscle protein synthesis and breakdown. However, both age-related sarcopenia and obesity are associated with the overexpression of myostatin, which functions as a protein inhibitor that negatively regulates skeletal muscle growth and homeostasis, as well as with the inhibition of myoblast proliferation and differentiation^10–12^. Previous trials have shown that resistance exercise training (RET) facilitates the initiation of muscle anabolism and increases the muscle protein synthesis rate in older adults^13–15^. Following RET, the net muscle protein balance, which denotes the difference between protein synthesis and breakdown, becomes less negative or even positive after protein supplementation^16^; this may further prevent skeletal muscle attenuation due to age. Hence, determining whether RET affects the loss of muscle mass in older adults with sarcopenic obesity is crucial.

RET has been used as an effective means of improving muscle function and increasing muscle mass in frail elderly people^3,14,17^. Resistance-type training using elastic bands has been frequently applied as a treatment method and is a safe approach for muscle strengthening in elderly adults^18,19^, with the results revealing muscle activations and self-perceived efficacy similar to those observed from free-weight resistance training^20^. Such training is used not only because of its simplicity and convenience but also its ability to strengthen the musculoskeletal system, which further benefits physical mobility^21,22^. In addition, an exercise program involving elastic tubing resistance training can provide more benefits compared with conventional free-weight resistance programs, such as increased functional strength, more effective injury prevention, greater ability to change muscle emphasis during exercises, and greater muscle power development^23^. Therefore, to help muscle strengthening with reduced burdens on joints, elastic tubing resistance training can be one of the most suitable resistive exercise programs for elderly people^19^.

Elastic band exercises are beneficial for elderly people. A study described the benefits of elastic resistance exercise involving. Theraband or tubing in preventing obesity or sarcopenia^24^. However, the effects of elastic band-based RET on muscle strength, body composition, and physical function (PF) in older women with sarcopenic obesity remain unclear. Therefore, in the present study, we investigated the effects of an elastic RET regime on muscle mass gain and PF in older women with this condition. In addition, we examined whether change in muscle mass was associated with physical capacity and function outcomes. We hypothesized that an elastic band RET regime could improve body composition and PF in older women with sarcopenic obesity.

## Results

**Figure 1:**
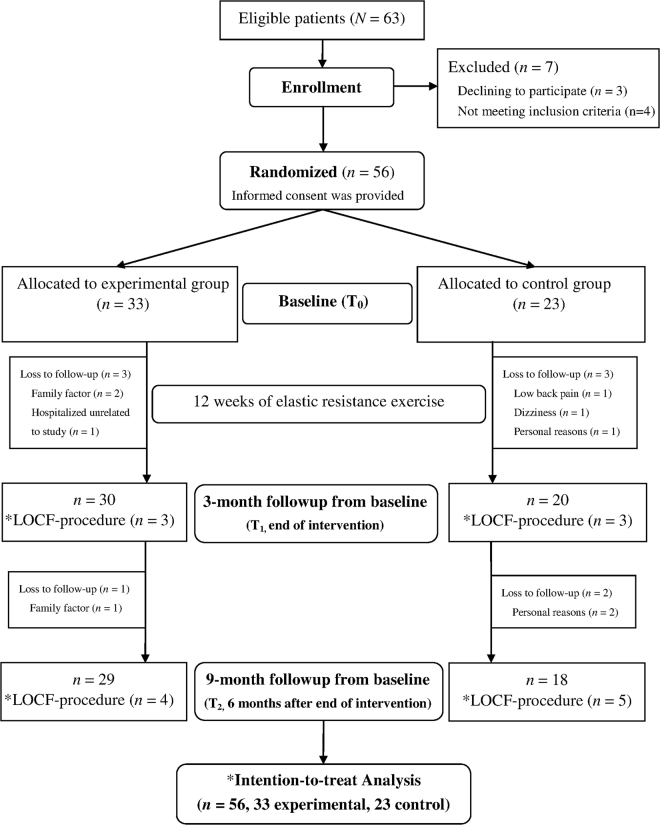
Flow diagram of patients throughout the present study.

Figure 1 depicts a flowchart of this study. A total of 63 patients were recruited. After 7 patients were excluded, 56 were enrolled and randomly allocated to either the experimental group (EG, n = 33) or control group (CG, n = 23) at baseline (T_0_). Subsequently, 30 patients in the EG and 20 patients in the CG completed the 3-month followup (T_1_). Moreover, 29 patients in the EG and 18 patients in the CG completed the 9-month followup assessments (T_2_). Based on the intention-to-treat principle, a total of nine pieces of missing data (six at T_1_ and three at T_2_) were imputed using the last-observation-carried-forward technique. At baseline, all patients were identified as sarcopenic, with a mean (SD) percentage skeletal muscle mass index (SMI) of 24.95% (2.48%), and obese with a percentage body fat (BF %) of 41.61% (7.34%). The majority of included patients (more than 80% in each group) were considered physically inactive based on self-reported participation levels in recreational physical or leisure activities (e.g., walking and running) of less than 1 h/wk within the previous 3 months. The compliance rate of the patients who participated in exercise interventions (i.e., EG) was 97.6% without any side effects being reported during their exercise period. Patients’ characteristics are presented in Table 1. An unequal distribution of baseline characteristics was noted despite no significant between-group differences being observed in patient characteristics. Therefore, we controlled for potential confounders between the two groups using age, comorbidities, and uneven baseline scores of measures as covariates in the statistical analysis.

**Table 1:** Patient demographic characteristics.

Item^a^	Control (*n* = 23)	Experimental (*n* = 33)	*P* value
	Mean±SD	Mean±SD	
Age (years)	68.32 ± 6.05	66.67 ± 4.54	0.276^d^
Height (cm)	151.68 ± 5.80	152.67 ± 5.85	0.537^d^
Weight (kg)	66.97 ± 8.31	63.45 ± 8.52	0.130^d^
BMI (kg/m^2^)	29.16 ± 3.62	27.27 ± 3.72	0.063^d^
CIRS	7.96 ± 1.85	7.45 ± 2.22	0.378^d^
PBF (%)^b^	43.71 ± 4.81	42.08 ± 3.48	0.147^d^
TSM (kg)^b^	16.91 ± 2.14	15.94 ± 2.18	0.705^d^
SMI (%)^b^	25.17 ± 2.19	24.80 ± 2.22	0.107^d^
Physically active, *n* (%)^c^	4(17.39)	5(15.15)	0.824^e^

^a^BMI = body mass index; CIRS = cumulative illness rating scale; PBF = percentage body fat; TSM = total skeletal muscle mass; SMI = appendicular skeletal mass index.

^b^Values were measured using bioelectrical impedance analysis.

^c^Defined as participation in a recreational physical or leisure activity (e.g., walking, running, hiking, swimming, ball exercise, gymnastics, bicycling, gardening) regularly (≥1 h/wk) within the past 3 months.

^d^Independent *t* test.

^e^Chi-squared test.

### Muscle mass outcome

Table 2 presents the adjusted mean change in the muscle mass and muscle index outcomes at T_1_ and T_2_. In the EG, the appendicular lean mass (ALM) at T_1_ and appendicular muscle index (AMI) at both T_1_ and T_2_ were significantly improved (all *P* < 0.05), compared with those at T_0_. In addition, after adjusting for baseline data, significant between-group differences in the absolute muscle mass variables and relative muscle mass indices were observed at T_1_ and T_2_. Compared with the CG, the EG exhibited significantly greater changes in total skeletal mass (TSM) at T_1_ and T_2_, with a mean difference (MD) of 0.70 kg (95% CI: 0.12–1.28; *P* < 0.05) and 0.72 kg (95% CI: 0.21–1.23; *P* < 0.01), respectively; similar results were observed for ALM, AMI, total lean mass index (LMI), and SMI (Table 2).

**Table 2:** Mean values of body composition and muscle indices at all time points and the changes from the baseline.

Measures	Time score^§^			*P* value^†^	Adjusted mean change from baseline^‡^			
	T_0_	T_1_	T_2_		T_1_ − T_0_	Difference (EG − CG)	T_2_ − T_0_	Difference (EG − CG)
	Mean(SD)	Mean(SD)	Mean(SD)		Mean(95% CI)	Mean(95% CI)	Mean(95% CI)	Mean(95% CI)
Body fat percentage^a^								
CG	43.40(5.23)	44.08(4.97)	43.99(4.52)	0.008	1.99(−0.37, 4.35)	−4.16(−7.28, −1.04)*	1.81(−0.54, 4.16)	−2.78(−5.89, 0.33)
EG	41.65(4.02)	40.89(3.77)	42.02(3.86)		−2.17(−4.13, −0.21)		−0.97(−2.92, 0.98)	
Absolute muscle mass^a^								
TSM (kg)								
CG	35.19(4.00)	34.75(3.56)	34.70(3.71)	0.007	−0.43(−0.88, 0.01)	0.70(0.12, 1.28)*	−0.49(−0.89, −0.11)	0.72(0.21, 1.23)**
EG	(34.784.14)	35.06(4.22)	35.00(4.24)		0.27(−0.09, 0.64)		0.22(−0.10, 0.55)	
ALM (kg)								
CG	13.97(1.87)	13.78(1.65)	13.56(1.56)	0.009	−0.26(−0.77, 0.24)	0.99(0.33, 1.66)**	−0.19(−0.60, 0.23)	0.49(−0.05, 1.04)
EG	14.21(2.03)	14.39(2.11)	14.26(2.20)		0.73(0.31, 1.15)		0.31(−0.03, 0.66)	
Relative muscle mass								
LMI (kg/m^2^)								
CG	15.31(1.62)	15.11(1.62)	15.22(1.47)	0.034	−0.19(−0.38, −0.03)	0.31(0.06, 0.56)*	−0.09(−0.29, 0.11)	0.05(−0.21, 0.31)
EG	14.94(1.79)	15.07(1.89)	15.01(1.85)		0.12(−0.04, 0.28)		0.04(−0.20, 0.13)	
AMI (kg/m^2^)								
CG	6.06(0.68)	6.16(0.73)	6.10(0.61)	0.047	0.02(−0.21, 0.25)	0.31(0.03, 0.61)*	0.01(−0.15, 0.16)	0.21(0.01, 0.42)*
EG	6.09(0.83)	6.37(0.76)^a^	6.23(0.92)		0.33(0.13, 0.52)		0.22(0.09, 0.35)	
SMI (%)								
CG	27.99(3.44)	27.69(4.00)	27.33(2.78)	0.039	−0.41(−1.36, 0.54)	0.69(−0.59, 1.97)	−1.04(−1.78, −0.30)	1.35(0.35, 2.34)**
EG	29.94(2.80)**	30.15(3.16)	29.98(2.93)		0.28(−0.50, 1.06)		0.30(−0.31, 0.91)	

Intention-to-treat analysis: *N* = 56 (experimental group *n* = 33 and control group *n* = 23).

*Significant difference compared with the control group, *P* < 0.05; **P < 0.01; ***P < 0.001.

^a^Values were measured using a dual-energy X-ray absorptiometer.

^¶^CG = control group; EG = experimental; TSM = total skeletal muscle mass; ALM = appendicular lean mass; AMI = appendicular lean mass index (appendicular lean mass/height^2^, kg/m^2^); LMI = lean mass index (whole body lean mass/height^2^, kg/m^2^); SMI = skeletal muscle mass index (whole body skeletal muscle mass/weight, %).

^**§**^T_0_ = baseline, T_1_ = follow-up at end of rehabilitation intervention, T_2_ = follow-up at 6 months after rehabilitation intervention.

^**†**^Repeated measure ANCOVA adjusted for age and comorbidity score.

^‡^All data were adjusted for age and comorbidity score. SMI was also adjusted for baseline score.

### Physical capacity and function outcomes

**Figure 2:**
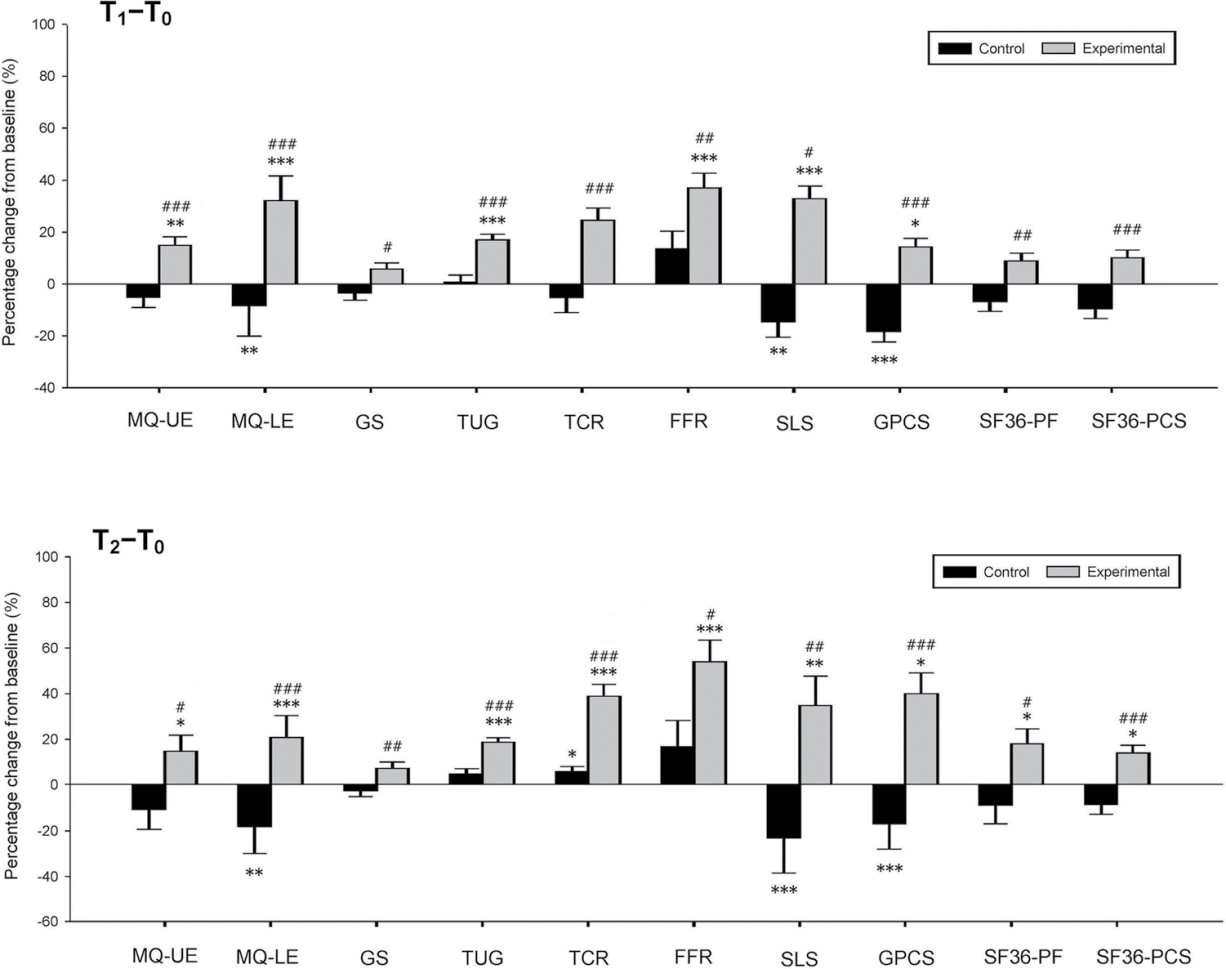
Changes in physical capacity and function outcomes at the 3-month follow-up (T_1_) and 9-month follow-up (T_2_) relative to the baseline (T_0_). The Y-axis represents the percentage change from the baseline. The error bar represents standard error. All data are presented as percentage change from the baseline and were adjusted for age, comorbidity score, body mass index, and body fat percentage at the baseline. *Significant difference as compared with the baseline, *P* < 0.05; **P < 0.01; ***P < 0.001. ^#^Significant difference compared with the control group, *P* < 0.05; ^##^*P* < 0.01; ^###^*P* < 0.001. MQ-UE = muscle quality of the upper extremity; MQ-LE = muscle quality of the lower extremity; GS = gait speed; TUG = timed up and go; TCR = timed chair rise; FFR = functional forward reach; SLS = single leg stance; GPCS = global physical capacity score; SF-36 PF = 36-item Short Form Health Survey, physical function subscore; SF-36 PCS = 36-item Short Form Health Survey, physical component summary.

Table 3 presents the mean values of the physical capacity and function outcome measures at various time points and the changes in values relative to the baseline. The changes in the physical capacity and function outcomes at T_1_ and T_2_ are also shown in Fig. 2. Significant between-group differences in the outcomes of all functional mobility tasks, muscle quality (MQ), and PF were observed at T_1_ and T_2_, with the difference in values in the various mobility and physical measures significantly exceeding the minimum clinically important difference (MCID) values (Table 3; Fig. 2). At T_1_, the EG walked faster by 0.14 m/s (*P* < 0.05), reached further by 7.46 cm (*P* < 0.001) in the functional forward reach (FFR) task, balanced for a longer time of 9.71 s (*P* < 0.001) in the single leg stance (SLS) test, spent a shorter time of 1.64 s (*P* < 0.001) in the Timed Up & Go (TUG) test, and completed 2.99 more repetitions (*P* < 0.001) of the timed chair rise (TCR) task than the CG did; similar results were observed at T_2_ (Table 3). Relative to the baseline, the global physical capacity score (GPCS) in the EG was significantly improved at T_1_ and T_2_, with adjusted changes of 1.34 (95% CI: 0.45–2.23; *P* < 0.05) and 1.96 (95% CI: 0.98–2.95; *P* < 0.001), respectively. Significant between-group differences were also obtained at T_1_ (MD 3.57, 95% CI: 2.16–4.97; *P* < 0.001) and T_2_ (MD 4.22, 95% CI: 2.67–5.77; *P* < 0.001) after adjustment for age and comorbidity score.

**Table 3:** Mean values of physical capacity and function outcome measures at the various time points and the changes from the baseline

Measures^¶^	Time score^§^			*P* value^†^	Adjusted mean change from baseline^‡^			Difference of change from the baseline (EG−CG)		
	T_0_	T_1_	T_2_		T_1_ − T_0_	T_2_ − T_0_	MDC_90_^a^	T_1_ − T_0_	T_2_ − T_0_	MCID^b^
	Mean(SD)	Mean(SD)	Mean(SD)		Mean(95% CI)	Mean(95% CI)	Value^reference^	Mean(95% CI)	Mean(95% CI)	Value^reference^
Physical capacity										
FRD (cm)							9.9^64^			NA
CG	33.92(11.02)	36.14(10.55)	36.19(10.41)	0.002	2.15(−0.84, 5.15)	2.63(−2.81, 8.07)		7.46(3.54, 11.38)***	8.66(1.53, 15.79)*	
EG	31.29(9.66)	40.87(9.61)	42.84(8.25)		9.61(7.13, 12.11)	11.29(6.76, 15.82)				
SLS (s)							23.6^64^			NA
CG	15.16(9.96)	12.08(8.99)	8.49(9.13)	< 0.001	−3.05(−6.04, −0.05)	−6.52(−10.15, −2.89)		9.71(5.79, 13.63)***	11.11(6.35, 15.87)***	
EG	14.27(10.47)	20.96(9.35)	18.96(9.87)		6.66(4.17, 9.16)	4.59(1.56, 7.61)				
GS (m/s)							0.1^73^			0.1^76^
CG	1.17(0.27)	1.16(0.20)	1.16(0.22)	0.008	−0.07(−0.15, −0.04)	−0.05(−0.12, 0.01)		0.14(0.33, 0.25)*	0.19(0.10, 0.28)***	
EG	1.44(0.28)**	1.46(0.24)	1.48(0.25)		0.07(0.01, 0.13)	0.13(0.08, 0.19)				
TUG (s)^c^							−2.5^73^			−1.2^77^
CG	9.45(2.38)	9.40(2.46)	9.06(2.48)	< 0.001	−0.02(−0.56, 0.51)	−0.36(−0.90, 0.18)		−1.64(−2.34, −0.95)***	−1.49(−2.21, −0.78)***	
EG	8.97(1.77)	7.32(1.27)	7.14(1.18)		−1.67(−2.11, −1.23)	−1.85(−2.31, −1.40)				
TCR (repetition)							1.6^67^			2.6^77^
CG	11.61(3.13)	11.83(3.14)	13.09(4.27)	< 0.001	−0.51(−1.68, 0.65)	0.48(−0.94, 1.89)		2.99(1.42, 4.56)***	4.16(2.25, 6.08)***	
EG	15.00(4.36)**	16.79(3.66)	18.94(3.18)		2.48(1.52, 3.44)	4.64(3.47, 5.80)				
GPCS							NA			NA
CG	11.61(3.63)	9.43(2.92)	8.91(2.58)	< 0.001	−2.23(−3.29, −1.16)	−2.25(−3.43, −1.07)		3.57(2.16, 4.97)***	4.22(2.67, 5.77)***	
EG	13.18(3.08)	14.48(2.84)	15.21(4.02)		1.34(0.45, 2.23)	1.96(0.98, 2.95)				
Muscle quality										
UE (kg/kg)							NA			NA
CG	11.60(3.31)	11.06(3.58)	11.13(3.41)	0.004	−1.68(−2.57, −0.80)	−0.92(−2.09, 0.26)		4.53(3.36, 5.69)***	3.88(2.31, 5.45)***	
EG	12.74(3.10)	14.21(3.18)	13.79(3.36)		2.85(2.11, 3.58)	2.96(1.99, 3.94)				
LE (N/kg)							NA			NA
CG	2.93(0.79)	2.48(0.66)	2.44(0.67)	< 0.001	−0.47(−0.91, −0.04)	−0.45(−0.89, 0.02)		1.82(1.25, 2.39)***	2.22(1.63, 2.81)***	
EG	2.48(.88)	3.81(1.37)	4.28(1.29)		1.35(0.99, 1.71)	1.78(1.40, 2.15)				
SF-36										
PF							27.9^74^			3.3^78^
CG	76.52(17.57)	74.20(19.21)	76.09(18.55)	0.003	−6.83(−12.71, −0.95)	−5.17(−10.43, 0.10)		13.00(5.03, 20.98)**	13.62(6.47, 20.76)***	
EG	91.11(13.38)**	94.14(11.27)	96.26(17.32)		6.17(1.36, 10.99)	8.45(4.13, 12.76)				
PCS							NA			2.0^78^
CG	68.87(17.12)	65.39(17.55)	65.91(18.38)	< 0.001	−7.51(−12.52, −2.49)	−6.76(−11.70, −1.82)		13.42(6.62, 20.22)***	15.06(8.36, 21.76)***	
EG	83.27(13.65)**	86.38(10.62)	88.93(10.14)		5.92(1.81, 10.02)	8.31(4.26, 12.35)				

Intention-to-treat analysis: *N* = 56 (experimental group *n* = 33 and control group *n* = 23).

*Significant difference compared with the control group, *P* < 0.05; **P < 0.01; ***P < 0.001.

^¶^CG = control group; EG = experimental group; FRD = functional reach distance; SLS = single leg stance; GS = gait speed; TUG = timed up and go test; TCR = timed chair rise; GPCS = global physical capacity score; UE = upper extremity; LE = lower extremity; SF-36 = 36-item Short Form Health Survey; PF = physical function; PCS = physical component summar y.

^**§**^T_0_ = baseline, T_1_ = followup at end of rehabilitation intervention, T_2_ = followup at 6 months after rehabilitation intervention.

^**†**^Repeated measure ANCOVA adjusted for age, comorbidity score, body mass index, and body fat percentage.

^‡^All data were adjusted for age and comorbidity score. GS, TCR, and SF-36 indices were also adjusted for baseline score.

^**a**^MDC_90_ = minimal detectable change at the 90% confidence interval; NA = data were not available from the literature.

^b^MCID = minimal clinical import difference; NA = data were not available from the literature.

^c^Negative value indicates an improvement from baseline.

At T_1_ and T_2_, the EG exhibited significantly greater increases in the Medical Outcomes Study Short Form-36 Questionnaire (SF-36) PF subscore, with MDs of 13.00 (95% CI: 5.03–20.98; *P* < 0.01) and 13.62 (95% CI: 6.47–20.76; *P* < 0.001), respectively, as well as in the physical component summary (PCS) score, with MDs of 13.42 (95% CI: 6.62–20.22; *P* < 0.001) and 15.06 (95% CI: 8.36–21.76; *P* < 0.001), respectively (Table 3; Fig. 2).

### Muscle mass change is associated with physical capacity and function outcomes

The Pearson correlation analysis showed that the changes in the muscle mass measures correlated with improvements in MQ, GPCS, and the SF-36 PF and PCS at T_2_ (Table 4). All of the muscle mass measures were entered stepwise into a linear regression model. Five iterations of multiple linear regression were performed to analyze the T_2_ data (Table 5), and the results indicated that a change in relative muscle mass (i.e., SMI and LMI) was associated with improved changes in physical outcome measures at T_2_. After controlling for group allocation, age, and comorbidity score, the adjusted change in relative muscle mass was significantly associated with improved MQ (*R*^2^ = 0.68, *P* < 0.001), GPCS (*R*^2^ = 0.55, *P* < 0.001), SF-36 PF (*R*^2^ = 0.35, *P* < 0.001), and SF-36 PCS (*R*^2^ = 0.36, *P* < 0.001).

**Table 4:** Correlations of changes in muscle mass measures and physical outcomes at the 9-month followup.

Muscle mass variable^¶**§**^	Muscle quality^¶^		Physical capacity^¶^						Physical function (SF‒36)^¶^	
	UE	LE	GS	TUG	TCR	FRD	SLS	GPCS	PF	PCS
ALM	0.629^**^	0.690^**^	0.188	−0.179	0.188	0.347^**^	0.125	0.561^**^	0.478^**^	0.475^**^
TSM	0.248	0.308^*^	0.112	−0.290^*^	0.217	0.428^**^	0.175	0.270^*^	0.197	0.408^**^
AMI	0.639^**^	0.730^**^	0.065	−0.095	0.048	0.093	0.015	0.588^**^	0.463^**^	0.501^**^
LMI	0.375^**^	0.350^**^	0.273^*^	−0.272^*^	0.076	0.182	0.149	0.340^*^	0.269^*^	0.534^**^
SMI	0.715^**^	0.787^**^	0.287^*^	−0.049	0.073	−0.310^*^	0.272^*^	0.693^**^	0.612^**^	0.500^**^

*P < 0.05, **P < 0.01, ***P < 0.001.

^¶^ALM = appendicular lean mass; TSM = total skeletal muscle mass; AMI = appendicular lean mass index (appendicular lean mass/height^2^, kg/m^2^); LMI = lean mass index (whole body lean mass/height^2^, kg/m^2^); SMI = skeletal muscle mass index (whole body skeletal muscle mass/weight, %); UE = upper extremity; LE = lower extremity; GS = gait speed; TUG = timed up and go test; TCR = timed chair rise; FRD = functional reach distance; SLS = single leg stance; GPCS = global physical capacity score; SF-36 = 36-item Short Form Health Survey; PF = physical function subscore; PCS = physical component summary.

^§^All muscle mass variables were transformed to percentage change from the baseline.

**Table 5:** Association of muscle mass changes with physical capacity and function outcomes at 6 months following resistance exercise intervention.

Covariate^¶§^	Model 1^a^		Model 2^b^	
	*β*	SE	*β*	SE
Dependent variable = MQ-UE				
SMI	4.125	0.599^***^	3.952	0.645^***^
LMI	2.250	1.110^*^	1.990	1.149
Adjusted *R*^2^	0.529		0.517	
Model *P* value	<0.001		<0.001	
Dependent variable = MQ-LE				
SMI	6.833	0.746^***^	6.161	0.747^***^
TSM	3.774	1.780^*^	2.496	1.805
Adjusted *R*^2^	0.636		0.678	
Model *P* value	<0.001		<0.001	
Dependent variable = GPCS				
SMI	5.989	0.847^***^	5.220	0.832^***^
Adjusted *R*^2^	0.471		0.554	
Model *P* value	<0.001		<0.001	
Dependent variable = SF-36 PF				
SMI	3.304	0.581^***^	3.278	0.626^***^
Adjusted *R*^2^	0.363		0.354	
Model *P* value	<0.001		<0.001	
Dependent variable = SF-36 PCS				
LMI	4.192	1.060^***^	4.117	1.116^**^
SMI	2.022	0.572^**^	1.844	0.626^**^
Adjusted *R*^2^	0.399		0.363	
Model *P* value	<0.001		<0.001	

*P < 0.05, **P < 0.01, ***P < 0.001.

^¶^SMI = skeletal muscle mass index (whole body skeletal muscle mass/weight, %); LMI = lean mass index (whole body lean mass/height^2^, kg/m^2^); TSM = total skeletal muscle mass; MQ-UE = muscle quality of the upper extremity; MQ-LE = muscle quality of the lower extremity; GPCS = global physical capacity score; SF-36 = 36-item Short Form Health Survey; PF = physical function subscore; PCS = physical component summary.

^§^All muscle mass variables were transformed to percentage change from the baseline.

^a^Model 1: Stepwise linear regression variables included muscle mass measures. The linear model coefficients are represented as the *β* values with standard error (SE).

^b^Model 2: Stepwise linear regression variables included group allocation, age, comorbidity score, and muscle mass measures from Model 1.

## Discussion

In this study, we investigated the effects of 12-week Theraband-based elastic resistance training on body composition and PF in older women with sarcopenic obesity. The results revealed that the EG had a significantly more favorable outcome for muscle mass and physical capacity (i.e., distance of FFR, time for TUG and SLS, effort of TCR, GS, and the SF-36 PF subscore) at T_1_ and T_2_, compared with the CG; in addition, the changes in muscle mass indices were independently associated with changes in PF outcome.

Unequal distribution of characteristics occurred at the baseline. Because of the unconstrained randomization procedure and the limited sample size, an equal distribution of baseline variables among the groups was not possible. Simple randomization is considered to have a probability of treatment imbalance, the potential for selection bias, and potential effects on power^25^. We therefore controlled for potential confounders between groups using baseline characteristics as covariates in our analysis. In addition, controlling for baseline differences in randomized controlled trials may improve the power without introducing bias^26,27^. All of these factors may explain the significant differences in muscle mass and physical outcome between the two groups.

Based on the fact that type-II myofiber atrophy is associated with aging and that RET predominantly benefits type-II myofiber hypertrophy, resistance-type exercises help elderly individuals with sarcopenia or obesity to counterforce muscle-attenuated physical difficulties through increasing muscle synthesis and muscle strength^3,14,17^. Because type-II myofibers have a recruitment pattern characteristic of high-threshold motor units, an intensity as high as 80%–95% of one repetition maximum (1RM) is required when setting an exercise intervention regarding type-II myofiber force production^28,29^. However, applying a heavy load ≥80% of 1RM is difficult for older populations, we considered the physiological stress related to the exercise load and set the exercise intensity based on the rating of perceived exertion (RPE) scale and RM load^29^. The exercise protocol was performed with a moderate-intensity on the RPE scale up to 13, which corresponded to a 60%–70% of 1RM load^30^, and was utilized with a 10RM and a 20RM exercise prescription. Our results were supported by the previous study demonstrating that intermediate RM load (9–11RM) exerts dramatically hypertrophic effects whereas high RM load (20–28RM) appears better adapted for submaximal and prolonged contractions^31^.

Based on the size principle, our training load did not provide an adequate hypertrophic stimulus for type-II motor unit recruitment^28^. However, previous studies had shown that older adults receiving variable resistance (i.e., 65% 1RM) exhibited as well increases in fat free mass as those receiving high resistance (i.e., 80% 1RM) did^32^; the results support our findings that patients receiving elastic RET had significant gains in muscle mass compared with those in the CG after the intervention. Our results further indicate that RET not only helps elderly people with sarcopenic obesity to improve their muscle mass but also to increase muscle strength, which may further overcame physical difficulties.

RET using elastic bands has recently been applied to obese^33–36^ and nonobese^18,21,22,24,37–39^ older adults safely and effectively^19^, regardless of the various protocols. Overall, relevant studies have used exercise protocols with an intervention period of 8–24 weeks, a frequency of 2–5 times per week, and a low to moderate-intensity. In general, studies have reported that body composition is improved through significant reductions in fat mass and increases in lean mass^33,35^, which also positively affect muscle structural change^18^, strength gain^18,22,33,35,39^, and functional mobility^24,33,34,36–39^. The results of relevant studies on elastic resistance exercise in elderly people support our findings concerning older individuals with sarcopenic obesity. Moreover, we determined that 12 weeks of moderate-intensity elastic RET exerted significant benefits on fat mass reduction, lean mass gain, strength gain, and functional mobility.

In this study, relative muscle mass indices (i.e., AMI, LMI, and SMI) generally showed greater correlations with improvements in PF measures, compared with absolute muscle mass variables (i.e., ASM and TLM); in addition, changes in relative muscle mass indices, rather than absolute muscle mass variables, were significantly associated with the improvement of physical outcomes. Sternfeld *et al*. reported a similar finding that absolute muscle mass was not significantly associated with functional mobility in older women^40^. Furthermore, in older obese people, low absolute skeletal muscle mass was not assumed to be associated with physical disability or mortality^41,42^. The implication of these observations is that interventions aimed at improving PF through changes in muscle mass may require a different emphasis in older women with sarcopenic obesity.

Our study has certain limitations. First, only female patients were included. Based on the sex-specific response to RET, our results might not be generalizable to all elderly populations. Second, neither lifetime physical activity nor lifestyle habits were specifically controlled; in addition, we did not objectively measure physical activity level using self-reported questionnaires such as the International Physical Activity Questionnaire or using measuring devices such as accelerometers. However, all participants were instructed to maintain their usual habits throughout the followup period. Third, we did not include an aerobic training program in our exercise regime, which mainly involved RET. The interventions combining aerobic training and RET for older adults have been reported to be more effective than those involving RET only for maintaining muscle mass^3^. However, the lean mass (or skeletal muscle mass) and muscle strength in older adults with obesity who received an RET-only exercise regime and in those who received a combination exercise regime (RET and aerobic exercise) have been reported as being comparable^43,44^. These findings justify our exercise protocol, which targeted the outcomes of muscle mass and function in older women with sarcopenic obesity.

Fourth, a discrepancy was observed between the numbers of participants in our experimental and control groups when the unrestricted randomization method was used. No significant differences were observed in the baseline characteristics of the participants between the two groups in this study, and potential confounders between the groups were controlled using baseline characteristics as covariates in the statistical analysis. However, our results were based on unequal group sizes, which may have introduced bias in the statistical analysis and may have consequently reduced the power of the study. Finally, we did not include a diet or nutrition supplement control during the exercise intervention. Therefore, we could not draw any conclusions regarding the relationship between nutrition supplements and changes in body composition during RET. Dietary patterns or nutrition supplements (e.g., protein) may interfere in changes to whole body weight or muscle mass during RET^45,46^.

In conclusion, this prospective study demonstrated that 12 weeks of elastic RET exerted positive benefits on muscle mass and functional capacity outcomes in older women with sarcopenic obesity. The results suggest that greater emphasis should be placed on elastic RET for muscle mass and strength gain in patients with sarcopenic obesity. The elastic RET protocol in and findings of this study can potentially assist clinician decision-making concerning the optimal treatment strategy for obese elderly women, particularly for those who are considered sarcopenic.

## Methods

### Study design

This prospective study was a randomized controlled trial conducted through a single-blind experimental design in a rehabilitation center at Shuang Ho Hospital, Taipei Medical University. All patients were enrolled from April 2015 to April 2016. After the patients provided consent, they were randomly assigned to one of two parallel groups: an EG receiving elastic RET and an age-matched CG. All outcome measures were assessed by a blinded examiner at the T_0_, at the end of the 12-week RET intervention (T_1_), and at 6 months following the completion of the RET intervention (T_2_).

### Ethics statement

This study was approved by the Joint Institutional Review Board of Taipei Medical University (trial number 201306019) and registered at the Chinese Clinical Trial Registry (trial number ChiCTR-IPR-15006069) on April 3, 2015. We confirm that the study methods adopted the CONSORT 2010 guidelines and were performed in accordance with the described procedures in the approved study protocol.

### Participants

Female patients aged from 60 to 80 years were recruited from the outpatient department of our rehabilitation center. Patients were included if they were identified as having sarcopenia or obesity. Patients were excluded if they had any of the following conditions: (a) Uncontrolled hypertension; (b) joint contractures or internal metal implants such as total joint arthroplasty; (c) cardiovascular or pulmonary diseases that could prevent them from engaging in an exercise study; or (d) neurological impairment.

### Definition of sarcopenic obesity

*Sarcopenia* Low muscle mass was determined using the same method as in our previous study^47^. First, total skeletal muscle mass (TSM, kg) was measured using an eight-polar bioelectrical impedance analysis device with multifrequency current (Inbody™ 220, Biospace, Seoul, Republic of Korea); this device was identified to be a valid TSM estimator^48^. Specifically, TSM was estimated using Janssen’s equation: TSM = [(Ht^2^/BIA-R) × 0.401 + (sex × 3.825) + (age × −0.071) + 5.102], where Ht represents the height in centimeters, BIA-R represents BIA resistance in ohms, and age is in years; moreover, for sex, men = 1 and women = 0^49^. The estimated TSM was converted to a percentage SMI through dividing it by total body mass in kilograms (TSM/total body mass × 100%). Patients with an SMI that was two standard deviations (SDs) lower than the sex-specific means of the younger population were considered to be sarcopenic. Because of the lack of a norm for the SMI among young adults in the local population, we used the reference value proposed by Janssen, which was derived from 3298 young women aged 18–39 years old. The cutoff value of the SMI for sarcopenia in women was less than 27.6%^47^.

*Obesity* Participants’ BF% was also measured through bioelectrical impedance analysis and was further used to determine obesity in this study. Patients with a BF% of more than 30% were considered to be obese^50^.

### Sample size

This study implemented lean mass gain as one of the key primary outcomes. The power calculation was based on the result of a previous similar study that compared elastic resistance-based exercise with modality-based rehabilitation in older female patients^51^. For an allowance to identify a significant between-group difference of 0.5 kg (assuming an SD of 2.86) on lean mass gain, 20 patients were required in each group, with a statistical power of 0.80, an effect size of 0.2, a correlation of 0.5 assumed for repeated measures, and an alpha value of 0.05. We anticipated a dropout rate of 20%, and thus included a total of 50 patients or more in the study. We also estimated the smallest sample size required for the multiple linear regression models established in our study. With a statistical power of 0.80, medium Cohen’s *f*^2^ effect size of 0.25^52,53^, alpha value of 0.05, and four predictors, our study required 53 patients to yield reliable results.

### Randomization

After the patients met the eligibility criteria and provided informed consent, we subsequently randomized patients using concealed allocation based on a list of random numbers, which was computer-generated by an independent randomization center. The principal investigator informed the patients about the treatment session schedules as allocated by the independent randomization center.

### Elastic resistance exercise

Resistance exercise was performed using Theraband (Hygenic Co., Akron, OH, USA). The color of the band denotes the degree of elasticity and the resistance level (yellow, red, green, blue, black, or silver). All patients undergoing elastic resistance exercise were trained and supervised by a licensed senior physical therapist who was blinded to the study group assignment. The Borg scale was used for RPE during training sessions; this is an efficient method for describing how the subjective intensity varies with physical intensity, and it further facilitates estimating the intensity of individual rehabilitation protocols^20,54^. Exercise loads in terms of individual yielding elasticity (as indicated by band color) in resistance training were set at a level that the patients perceived as “somewhat hard,” which is equivalent to a 13-grade rating (representing a moderate-intensity exercise) on the RPE scale, according to the American College of Sports Medicine^55^. Exercise movements were designed on the basis of previously established elastic exercise regimes used for training older female adults;^33,38,56^ the designed movements were aimed at strengthening the main muscle groups in the trunk, upper extremities, and lower extremities that are crucial for physical mobility^33,56,57^. For each exercise movement, 3 sets of 10 repetitions of gentle concentric and eccentric contractions through the full range of motion were slowly performed using a yellow Theraband initially (Supplementary Appendix S1). The exercise intensity was increased when the patients were able to achieve a perceived yield strength corresponding to a 13-grade rating on the RPE scale. If the progressively advancing exercise load level could not be managed (i.e., from red to green Theraband), the previously used Theraband color was maintained with an additional set of each exercise motion until the patients could yield that exercise load.

### Training protocol

The training protocol was designed on the basis of the ACSM guidelines for resistance training with older subjects^55^. Each patient in the EG underwent 3 training sessions every week for 12 weeks, yielding a total of 36 sessions within 3 months (Supplementary Appendix S1). Each training session comprised a 10-min warm-up, 40-min period of elastic resistance exercises, and 5-min cool-down period; the session was supervised by a senior licensed physical therapist. After the warm-up, patients performed upper body exercises (seated chest press, seated row, and seated shoulder press) followed by lower body exercises (knee extension, knee flexion, hip flexion, and hip extension). The detailed exercise regimen is presented in Supplementary Appendix S2.

### Muscle mass assessment

Muscle mass was measured using a Hologic QDR-1000/W whole body dual-energy X-ray absorptiometer (Hologic, Waltham, MA, USA). All scans and analyses were conducted by the same investigator, who was blinded to the patients’ group allocation to minimize interobserver variation. Hologic enhanced whole-body analysis software (version 5.71) was used to provide estimates of the following components: whole body lean mass (kg), TSM (kg), and ALM (kg). According to the recommendations of the Asian Working Group for Sarcopenia and the European Working Group on Sarcopenia in Older People, three relative muscle mass indices were adopted to define muscle mass outcomes^58,59^. The first two major indices, namely AMI (kg/m^2^) and LMI (kg/m^2^), are calculated as the ALM and whole body lean mass divided by squared height in meters, respectively. The third major index was SMI (%) which as mentioned above.

### Muscular strength measurement

In this study, muscle strength was assessed in terms of MQ, which is defined as the ratio of muscular strength to muscle mass, and it was reported to be an indicator of muscle function^60,61^. The MQ of the upper extremity is calculated by dividing handgrip strength (kg) by arm lean mass (kg), whereas that of the lower extremity is calculated by dividing the strength of isometric quadriceps (N) by leg lean mass (kg).

#### Upper extremity strength

Handgrip strength was measured using a standard hydraulic hand dynamometer (Baseline^®^ Digital, Fabrication Enterprises Inc., New York City, NY, USA). Each patient’s dominant hand was tested. Patients were seated with their arms adducted, the tested forearm unsupported, the elbow flexed at an angle of 90°, and the wrist in a neutral position. The width of the dynamometer handle was adjusted to allow the middle phalange of the third digit to be comfortably perpendicular to the long axis of the handle. All patients were asked to perform a maximal contraction by squeezing the dynamometer handle as forcefully as possible for 3–5 s, with an encouraging verbal cue being provided during this task. Three trials were performed, with a rest period between trials of approximately 30 s. The force output (kg) was recorded for each trial, and the average of the three trials was considered as the representative handgrip strength value^46^.

#### Lower extremity strength

Measurement of maximal isometric quadriceps strength in the dominant leg adopted Buckinx’s method^62^, using a handheld dynamometer (Microfet3; Hoggan Health Industries Co., UT, USA). Each patient was seated in a chair with the knee and hip flexed at 90° and pelvis fixed. The dynamometer pad was placed just proximal to the lateral malleolus. All patients were asked to make their greatest effort to extend the leg against the dynamometer for 10 s. Three trials were performed, with a rest period between trials of approximately 30 s. Maximal force output (N) was recorded for each trial, and the average of the three trials was considered as the representative knee extensor strength.

Studies have reported that conducting the isometric strength test in older adults using a handheld dynamometer provided excellent reliability, with an intraclass correlation coefficient (ICC) ranging from 0.97 to 0.98 for handgrip strength^63^ and an ICC of 0.81 [95% confidence interval (CI): 0.68–0.93] for knee extensor strength^62^.

### Physical capacity assessment

Physical capacity of patients was assessed using the physical performance battery of mobility tasks. All physical mobility tasks used in the present study have been validated in older adults^64–68^. Each patient performed a practice trial before the test, and two trials were performed. The mean score of the two tested trials of each task was calculated for statistical analysis.

#### Functional forward reach

We used an FFR task to assess balance performance. The FFR task provides a dynamic measure of balance regardless of the selected movement strategy^69^.

#### Single leg stance

SLS tasks were used to assess balance control. The SLS score represents the total time for which a patient can stand on the dominant leg. SLS tasks were performed separately with eyes open and closed.

#### Gait speed (GS)

The GS was measured using the time required for the patient to walk 10 m on a track at a self-determined pace^65^. The GS was calculated in meters per second for each patient.

#### Timed Up & Go test

The TUG test measures the time required for a patient to rise from a chair (height 42 cm, depth 26 cm), walk 3 m, turn around, and return to a seated position in the chair at a self-determined speed. A walking aid was used by patients during the test if necessary.

#### Timed chair rise

In the TCR assessment, patients were asked to stand upright from a seated position in a chair (height 43 cm) with their arms folded across their chest and return to a seated position as many times as possible within a 30-s period.

#### Global physical capacity score

The GPCS used in the present study was adapted from approaches in relevant studies^9,70^. Each of the five mobility tests (FFR, SLS, GS, TUG, and TCR) was assigned a score from 1 to 4 using quartiles of performance (i.e., fourth, third, second, and first quartiles were coded as 4, 3, 2, and 1 points, respectively). Patients who could not complete the test were assigned a score of 0. Scores for all tests were summed to obtain a GPCS for each patient (possible score range: 0–20).

Compared with each individual test, one advantage of GPCS is that it provides an overall measure of patient performance through considering several tasks related to daily activities. Consequently, we used the GPCS to identify whether patients with sarcopenic obesity receiving RET had a higher functional capacity than did the CG.

#### Medical Outcomes Study Short Form-36 Questionnaire (SF-36)

Health-related quality of life was assessed using the Chinese version of the Medical Outcomes Study SF-36^71^. It comprises 36 items divided into 8 domains for which a subscale score is calculated. From the SF-36, four subscales (i.e., PF, role physical, bodily pain, and general health) are incorporated into a summary measure called the PCS. The domains and PCS are transformed into a score from 0 to 100, where 100 indicates no symptoms and 0 indicates extreme symptoms. Good validity (ICC = 0.72–0.88) and reliability (ICC = 0.66–0.94) has been established for the Chinese version of the SF-36^72^.

The well-established values of minimum detectable change at a 90% level of confidence (MDC_90_) of the outcome measures used in the present study (i.e., handgrip, isometric quadriceps strength, FFR, SLS, GS, and SF-36 PF subscore) for older adults are available in relevant studies^64,67,73,74^, or can be estimated using the formula MDC_90_ = z × SEM × 2^½^, where z is 1.65 at a 90% CI^75^ and SEM is the standard error of the mean. All available MDC_90_ values are presented in Table 3.

### Statistical analysis

Independent *t*-tests and chi-squared analyses were used to compare the patient characteristics and outcome measures of the EG with those of the CG at the baseline. The Kolmogorov–Smirnov test was used to confirm whether all variables were normally distributed. An intent-to-treat analysis based on the last-observation-carried-forward technique was used to manage any missing data and minimize bias related to losses during followup. A 2 × 3 repeated measures analysis of covariance (ANCOVA) was used to analyze all outcome variables. In the ANCOVA, time served as the repeated measures factor (T_0_, T_1_, and T_2_), and the allocated group (RET vs. control) served as the between-groups factor. In addition, the baseline score for each outcome measure was considered a potentially confounding covariate to improve the precision of the effect estimates. All comparison results that had *P* < 0.05 were considered statistically significant, and are presented as mean values with SDs. The SPSS statistical software package for Windows (Version 17.0. Chicago: SPSS Inc) was used for all analyses.

To test whether the intragroup changes were clinically significant, we compared the MCID of each outcome measure with either the 95% CI or the percentage of postintervention change. The following established MCIDs for the outcome measures of older adults were obtained from the literature: 0.1 m/s for GS^76^; 2.0–2.6 repetitions for the TCR^77^; 1.2 s for the TUG^77^; and 3.3 and 2.0 for SF-36 PF and PCS, respectively^78^.

When controlling for the exercise intervention mode and baseline characteristics, we determined the associations between the changes in muscle mass and those in physical outcomes at T_2_ through conducting a multiple stepwise linear regression analysis on the collapsed data. In these data, the changes in the physical outcome measures (i.e., the MQ of the upper and lower extremity, GPCS, SF-36 PF, and SF-36 PCS) were treated as dependent variables, and the changes in the muscle mass measures (i.e., the ALM, TSM, AMI, LMI, and SMI) were treated as exploratory covariates.

### Chinese Clinical Trial Registration Numbers

ChiCTR-IPR-15006069, www.chictr.org.cn, Registration date: April 03, 2015.

## Electronic supplementary material

Supplementary Appendices
