# Supplementary material for: Effects of elastic band exercise on lean mass and physical capacity in older women with sarcopenic obesity: A randomized controlled trial
Source: Sci Rep. 2018 Feb 2;8:2317. doi: 10.1038/s41598-018-20677-7 (PMC5797161; doi:10.1038/s41598-018-20677-7)
Supplement: Supplementary file 1 — Supplementary Appendices [file 41598_2018_20677_MOESM1_ESM.pdf]

Effects of elastic band exercise on lean mass and physical capacity in older women with sarcopenic obesity: A randomized controlled trial

Chun-De Liao, Jau-Yih Tsauo, Shih-Wei Huang, Jan-Wen Ku, Dun-Jen Hsiao, Tsan-Hon Liou

Supplementary Appendix S1

Exercise progression protocol

	Week	**1**	**2**	**3**	**4**	**5**	**6**	**7**	**8**	**9**	**10**	**11**	**12**
Theraband color	Yellow	X	X										
	Red			X	X								
	Green					X	X						
	Blue							X	X				
	Black									X	X		
	Silver											X	X
Exercise loading	Repetition	10	20	10	20	10	20	10	20	10	20	10	20
	Set	3	3	3	3	3	3	3	3	3	3	3	3
	RPE[a]	10 − 13	10 − 13	10 − 13	10 − 13	10 − 13	10 − 13	10 − 13	10 − 13	10 − 13	10 − 13	10 − 13	10 − 13

X = the used theraband colour

[a]Ratings of perceived exertion according to the Borg scale

Supplementary Appendix S2

Elastic-band resistance exercise regime

Movement	Intensity (repetition/set)	Targeted muscle group	Duration (min)
**A. Warm-up**			
1. Mobility exercise of the neck, upper limbs, and back		Upper quarter flexors and extensors	5
2. Global flexion-extension of the lower limb		Lower quarter flexors and extensors	5
**B. Upper quarter**			
1. Seated chest press	10–20/3	Upper quarter extensors	5 – 10
2. Seated row	10 – 20/3	Upper quarter flexors	5 – 10
3. Seated shoulder press	10 – 20/3	Shoulder girdle muscle groups	5 – 10
**C. Lower quarter**			
1. Concentric–eccentric hip circumduction	10 – 20/3	Hip girdle muscle groups	5 – 10
2. Leg press	10 – 20/3	Lower quarter extensors	5 – 10
3. Leg curl	10 – 20/3	Lower quarter flexors	5 – 10
**D. Cool down**			5
